# Supplementary material for: Impact of the Immunomodulatory Factor Soluble B7-H4 in the Progress of Preeclampsia by Inhibiting Essential Functions of Extravillous Trophoblast Cells
Source: Cells. 2024 Aug 17;13(16):1372. doi: 10.3390/cells13161372 (PMC11352994; doi:10.3390/cells13161372)
Supplement: Supplementary file 1 [file cells-13-01372-s001.zip › cells-3103069-supplementary.pdf]

Supplementary material: Ma, Duan et al.

Supplementary Table S1. Clinical characteristics of pregnant women and newborns in preterm control group, early-onset PE and PE+TPE group

Variable	Control (*n* = 12)	PE (*n* = 13)	PE + TPE (*n* = 12)	*p* value
Maternal age at delivery, years, mean ± SD	32.92 ± 6.36	32.15 ± 4.58	30.92 ± 6.24	0.6937
Gestational age at delivery, weeks, mean (min-max)	30 (22+4-33+6)	27+3 (23+5 -30)	25+3 (23+2 -28+5)	0.0060#
Pregnancy BMI before birth, mean ± SD	30.70 ± 7.73	30.30 ± 7.57	33.81 ± 8.65	0.5505
Cesarean section, no. (%)	75	100	100	nm
Systolic blood pressure, mmHg, mean ± SD	118.90 ± 14.43	154.40 ± 25.14	160.50 ± 15.69	<0.0001†, #
Diastolic blood pressure, mmHg, mean ± SD	67.92 ± 11.54	90.62 ± 18.31	88.67 ± 11.69	0.0010†, #
Proteinuria, mg/24 hour, mean ± SD	nm	1672 ± 2099	2908 ± 1888	0.1145
Platelet count, cells/mm3, mean ± SD	267.3 ± 68.06	172.0 ± 80.12	179.8 ± 67.57	0.0096†, #
Serum B7-H4, ng/ml, mean ± SD	5.362	20.10 ± 18.93	12.02 ± 9.99	nm
Birth weight, g, mean ± SD	1437.0 ± 677.5	1030.0 ± 778.2	552.7 ± 258.7	0.0027#

Abbreviations: BMI, body mass index; nm, not measured.
Notes: † denotes $p < 0.05$ between control and non-TPE-treated group; # means $p < 0.05$ between control and TPE-treated group; § means $p < 0.05$ between non-TPE-treated and TPE-treated group.
